# Vitamins A, C and E and the risk of breast cancer: results from a case-control study in Greece

**DOI:** 10.1038/sj.bjc.6690006

**Published:** 1999-09-24

**Authors:** K Bohlke, D Spiegelman, A Trichopoulou, K Katsouyanni, D Trichopoulos

**Affiliations:** Department of Epidemiology, Harvard School of Public Health,677 Huntington Avenue, Boston, MA, 02115, USA; Department of Nutrition and Biochemistry, Athens School of Public Health, Leoforos Alexandras 196, Athens, 115–21, Greece; Department of Hygiene and Epidemiology, University of Athens Medical School, Goudi, Athens, 115–27, Greece

**Keywords:** breast neoplasms, diet, β-carotene, retinol, vitamin C, vitamin E, Greece

## Abstract

Although several dietary compounds are hypothesized to have anticarcinogenic properties, the role ofpecific micronutrients in the development of breast cancer remains unclear. To address this issue, we assessed intake of retinol, β-carotene, vitamin C and vitamin E in relation to breast cancer risk in a case–control study in Greece. Eight hundrednd twenty women with histologically confirmed breast cancer were compared with 1548 control women. Dietary data were collectedhrough a 115-item semiquantitative food frequency questionnaire. Data were modelled by logistic regression, with adjustment forotal energy intake and established breast cancer risk factors, as well as mutual adjustment among the micronutrients. Amongost-menopausal women, there was no association between any of the micronutrients evaluated and risk of breast cancer. Amongremenopausal women, β-carotene, vitamin C and vitamin E were each inversely associated with breast cancer risk, but afterutual adjustment among the three nutrients only β-carotene remained significant; the odds ratio (OR) for a one-quintilencrease in β-carotene intake was 0.84 (95% confidence interval 0.73–0.97). The inverse association observed with β-carotene intake, however, is slightly weaker than the association previously observed with vegetable intake in these data,aising the possibility that the observed β-carotene effect is accounted for by another component of vegetables. ©1999 Cancer Research Campaign


*British Journal of Cancer* (1999) **79**(1), 23–29
© 1999 Cancer Research Campaign

### Vitamins A, C and E and the risk of breast cancer: results from a case–control study in Greece

**K Bohlke[1], D Spiegelman[1], A Trichopoulou[2], K Katsouyanni[3] and D Trichopoulos[1]**

[1]Department of Epidemiology, Harvard School of Public Health, 677 Huntington Avenue, Boston, MA 02115, USA; [2]Department of Nutrition and Biochemistry, Athens School of Public Health, Leoforos Alexandras 196, Athens 115–21, Greece; [3]Department of Hygiene and Epidemiology, University of Athens Medical School, Goudi, Athens 115–27, Greece

**Summary** Although several dietary compounds are hypothesized to have anticarcinogenic properties, the role of specific micronutrients in the development of breast cancer remains unclear. To address this issue, we assessed intake of retinol, β-carotene, vitamin C and vitamin E in relation to breast cancer risk in a case–control study in Greece. Eight hundred and twenty women with histologically confirmed breast cancer were compared with 1548 control women. Dietary data were collected through a 115-item semiquantitative food frequency questionnaire. Data were modelled by logistic regression, with adjustment for total energy intake and established breast cancer risk factors, as well as mutual adjustment among the micronutrients. Among post-menopausal women, there was no association between any of the micronutrients evaluated and risk of breast cancer. Among premenopausal women, β-carotene, vitamin C and vitamin E were each inversely associated with breast cancer risk, but after mutual adjustment among the three nutrients only β-carotene remained significant; the odds ratio (OR) for a one-quintile increase in β-carotene intake was 0.84 (95% confidence interval 0.73–0.97). The inverse association observed with β-carotene intake, however, is slightly weaker than the association previously observed with vegetable intake in these data, raising the possibility that the observed β-carotene effect is accounted for by another component of vegetables.

**Keywords:** breast neoplasms; diet; β-carotene; retinol; vitamin C; vitamin E; Greece

In the search for modifiable determinants of breast cancer, dietary factors are an ongoing focus of research. Migrant studies (Buell, 1973; Thomas and Karagas, 1987; Shimizu et al, 1991; Ziegler et al, 1993) support the hypothesis that diet or other lifestyle factors may partially explain the international differences in breast cancer incidence, and experimental data demonstrate that dietary factors may enhance or inhibit mammary carcinogenesis in animals (Tannenbaum, 1942; Albanes, 1987). Dietary factors explored as potentially protective for breast cancer include vitamins A, C and E. Vitamin A, from retinol (preformed vitamin A) or provitamin A carotenoids, plays a role in epithelial cell differentiation and control of proliferation (Sporn and Roberts, 1983). In addition, carotenoids act as antioxidants (Peto et al, 1981; Ames, 1983), as do vitamin C and vitamin E (Ames, 1983).

The epidemiological evidence regarding intake of these micronutrients and risk of breast cancer is strongest for carotenoids and vitamin C, although for both nutrients the associations observed in case–control studies have generally been stronger than the results observed in cohort studies. Inverse associations with vitamin E have also been observed in a number of case–control studies, but most prospective studies have reported null findings. Results for retinol intake have also been mixed. These and other nutrients have been reviewed by Hunter and Willett (1996).

The present study assesses the intake of retinol, β-carotene, vitamin C and vitamin E in relation to the risk of breast cancer in a case–control study in Greece. A question of interest is the extent to which any association observed with these micronutrients can be attributed to the nutrients per se rather than to other unmeasured components of fruits and vegetables.

*Received 5 January 1998*
*Revised 13 April 1998*
*Accepted 20 April 1998*

*Correspondence to:* K Bohlke, Group Health Cooperative Center for Health Studies, 1730 Minor Ave, Suite 1600, Seattle, WA 98101, USA

### MATERIALS AND METHODS

During a 3-year period from January 1989 until December 1991, all women with newly diagnosed breast cancer who were residents of the greater Athens area (Athens, Piraeus and surroundings; population about 3.5 million) were identified from four major hospitals. These four hospitals account for approximately 50% of all breast cancer cases occurring in this area. Of a total of 873 histologically confirmed cases, 820 (94%) were successfully interviewed and included in this study. All interviews took place in the hospital before the first discharge.

Two controls were selected for each case, one from among hospital visitors to the same hospital and one from among orthopaedic patients at the major accident hospital in Athens (for breast cancer cases residing in or around Athens) or Piraeus (for breast cancer cases residing in or around Piraeus). Controls were selected from among women of the same age (within 5 years) and residential area as the index case. A total of 808 eligible visitor controls and 830 eligible orthopaedic patient controls were identified; of these, 753 (93%) of the visitor controls and 795 (96%) of the orthopaedic patient controls were interviewed and included in the study. Control interviews were conducted in the hospital by the same interviewer who interviewed the index case. Additional information regarding subject selection has been presented elsewhere (Katsouyanni et al, 1994*a*).

The interview included demographic, socioeconomic, reproductive and biomedical variables, as well as a semiquantitative food

**Table 1** Distribution of 820 breast cancer cases and 1548 controls[a] by demographic and reproductive variables[b]

| | Cases | Controls |
|---|---|---|
| Age (years) | 56.4 (0.43)[c] | 54.4 (0.32) |
| Place of birth | | |
| Urban | 620 (75.7) | 1106 (71.6) |
| Rural | 199 (24.3) | 439 (28.4) |
| Age at menarche (years) | 12.9 (0.06) | 13.1 (0.04) |
| Quetelet index (kg m$^{-2}$) | 26.6 (1.02) | 25.9 (0.75) |
| Ever pregnant | | |
| Yes | 657 (80.2) | 1164 (75.2) |
| No | 162 (19.8) | 384 (24.8) |
| Age at first birth (years) | 26.4 (0.21) | 25.9 (0.16) |
| Menopausal status | | |
| Post-menopausal | 550 (67.1) | 1041 (67.3) |
| Premenopausal | 270 (32.9) | 505 (32.7) |

[a]There are a few missing values. [b]Adapted from Katsouyanni et al (1994*a*). [c]Numbers in parentheses are standard errors for quantitative variables and percentages for qualitative variable.

frequency questionnaire. For the food frequency questionnaire, subjects were asked to indicate their average intake of 115 food items per day, week or month during the year before the present disease (or the interview for visitor controls). A version of this questionnaire has been validated (Gnardellis et al, 1995; Katsouyanni et al, 1997). For the purpose of analysis, the intake of each food item was quantified approximately as the number of times the food was consumed per month, as it was by Graham et al (1978) and Katsouyanni et al (1991*a*). Thus, daily intake was multiplied by 30 and weekly intake by 4; food items consumed rarely or never were given a value of 0.

Intake of specific nutrients was estimated by multiplying the nutrient content of a selected typical portion of each food item by the frequency the food item was eaten per month and summing over all food items. Food consumption data were based on a nutrient database developed in Greece by the Department of Nutrition and Biochemistry, Athens School of Public Health (Trichopoulou, 1992). The portion size estimation was based on the results from previous validation studies (Katsouyanni et al, 1991*b*; Gnardellis et al, 1995), and the nutrient content was calculated on the basis of Greek recipes (Trichopoulou, 1992). Vitamin supplement use was very uncommon in this population. Because of the positive correlation between intake of most nutrients and total energy intake, multivariate models include total energy intake as a potential confounder. Analyses of fat and other macronutrients, alcohol intake and specific food groups have been presented in earlier reports (Katsouyanni et al, 1994*a*, 1994*b*; Trichopoulou et al, 1995), but the associations, if any, with micronutrients such as vitamins A and C have not been previously examined.

Results were modelled by unconditional logistic regression (Breslow and Day, 1980) using the SAS statistical package (SAS Institute, Cary, NC, USA). Although cases and controls were paired with respect to age, place of residence and interviewer, this was done essentially for administrative purposes, to facilitate enrolment of cases and controls in an operationalized way that

**Table 2** Frequency distribution of breast cancer cases and controls by quintile of intake[a] of selected micronutrients, among all women and stratified by menopausal status[b]

| Micronutrient | All women | | Premenopausal women | | Post-menopausal women | |
|---|---|---|---|---|---|---|
| | Cases (%) | Controls (%) | Cases (%) | Controls (%) | Cases (%) | Controls (%) |
| Retinol (µg day$^{-1}$) | | | | | | |
| ≤659.1 | 159 (19.4) | 310 (20.0) | 34 (12.6) | 69 (13.7) | 125 (22.8) | 241 (23.2) |
| 659.2–1306.3 | 169 (20.6) | 309 (20.0) | 47 (17.4) | 79 (15.6) | 122 (22.2) | 228 (21.9) |
| 1306.4–1575.2 | 170 (20.8) | 309 (20.0) | 55 (20.4) | 100 (19.8) | 115 (21.0) | 209 (20.1) |
| 1575.3–2120.7 | 155 (18.9) | 309 (20.0) | 60 (22.2) | 115 (22.8) | 95 (17.3) | 194 (18.7) |
| >2120.7 | 166 (20.3) | 310 (20.0) | 74 (27.4) | 142 (28.1) | 92 (16.8) | 168 (16.2) |
| β-Carotene (µg day$^{-1}$) | | | | | | |
| ≤3780.7 | 195 (23.8) | 309 (20.0) | 62 (23.0) | 83 (16.4) | 133 (24.2) | 225 (21.6) |
| 3780.8–5185.6 | 178 (21.7) | 310 (20.0) | 52 (19.3) | 89 (17.6) | 126 (23.0) | 221 (21.3) |
| 5185.7–6465.4 | 143 (17.5) | 310 (20.0) | 59 (21.9) | 97 (19.2) | 84 (15.3) | 212 (20.4) |
| 6465.5–8362.4 | 154 (18.8) | 309 (20.0) | 52 (19.3) | 114 (22.6) | 102 (18.6) | 195 (18.8) |
| >8362.4 | 149 (18.2) | 309 (20.0) | 45 (16.7) | 122 (24.2) | 104 (18.9) | 187 (18.0) |
| Vitamin C (mg day$^{-1}$) | | | | | | |
| ≤142.9 | 162 (19.8) | 310 (20.0) | 41 (15.2) | 70 (13.9) | 121 (22.0) | 240 (23.1) |
| 143.0–212.0 | 203 (24.8) | 310 (20.0) | 65 (24.1) | 94 (18.6) | 138 (25.1) | 215 (20.7) |
| 212.1–274.0 | 163 (19.9) | 309 (20.0) | 59 (21.9) | 90 (17.8) | 104 (18.9) | 218 (20.9) |
| 274.1–343.1 | 151 (18.4) | 310 (20.0) | 52 (19.3) | 110 (21.8) | 99 (18.0) | 200 (19.2) |
| >343.1 | 140 (17.1) | 309 (20.0) | 53 (19.6) | 141 (27.9) | 87 (15.9) | 168 (16.1) |
| Vitamin E (IU day$^{-1}$) | | | | | | |
| ≤5.2 | 171 (20.9) | 310 (20.0) | 33 (12.2) | 58 (11.5) | 138 (25.1) | 251 (24.1) |
| 5.3–6.2 | 174 (21.3) | 309 (20.0) | 55 (20.4) | 96 (19.0) | 119 (21.7) | 213 (20.5) |
| 6.3–7.2 | 165 (20.2) | 310 (20.0) | 66 (24.4) | 91 (18.0) | 99 (18.0) | 218 (21.0) |
| 7.3–8.6 | 140 (17.1) | 308 (19.9) | 60 (22.2) | 127 (25.2) | 80 (14.6) | 181 (17.4) |
| >8.6 | 169 (20.6) | 310 (20.0) | 56 (20.7) | 133 (26.3) | 113 (20.6) | 177 (17.0) |

[a]Quintiles are based on the control distribution. [b]There are a few missing values.

 © Cancer Research Campaign 1999

**Table 3** Multiple logistic regression derived odds ratios (OR) and 95% confidence intervals (CI) for the associations between quintile of nutrient intake and breast cancer, among all women and stratified by menopausal status

| Nutrient | All women | | Premenopausal women | | Post-menopausal women | |
|---|---|---|---|---|---|---|
| | OR[a] | 95% CI | OR[b] | 95% CI | OR[b] | 95% CI |
| Retinol (μg day$^{-1}$) | | | | | | |
| ≤659.1 | 1.00[c] | – | 1.00 | – | 1.00 | – |
| 659.2–1306.3 | 0.99 | (0.73–1.33) | 1.26 | (0.71–2.26) | 0.89 | (0.63–1.27) |
| 1306.4–1575.2 | 1.03 | (0.76–1.39) | 1.28 | (0.73–2.24) | 0.91 | (0.64–1.31) |
| 1575.3–2120.7 | 0.84 | (0.61–1.15) | 1.18 | (0.67–2.05) | 0.68 | (0.46–1.01) |
| >2120.7 | 0.89 | (0.65–1.23) | 1.16 | (0.67–2.01) | 0.80 | (0.53–1.21) |
| *P*-value for trend[d] | 0.31 | | 0.83 | | 0.14 | |
| β-carotene (μg day$^{-1}$) | | | | | | |
| ≤3780.7 | 1.00 | – | 1.00 | – | 1.00 | – |
| 3780.8–5185.6 | 0.83 | (0.62–1.12) | 0.67 | (0.40–1.13) | 0.94 | (0.66–1.34) |
| 5185.7–6465.4 | 0.70 | (0.52–0.95) | 0.73 | (0.44–1.20) | 0.65 | (0.44–0.95) |
| 6465.5–8362.4 | 0.70 | (0.52–0.95) | 0.49 | (0.29–0.83) | 0.83 | (0.57–1.21) |
| >8362.4 | 0.67 | (0.49–0.91) | 0.36 | (0.21–0.61) | 0.90 | (0.61–1.35) |
| *P*-value for trend | 0.005 | | 0.0001 | | 0.38 | |
| Vitamin C (mg day$^{-1}$) | | | | | | |
| ≤142.9 | 1.00 | – | 1.00 | – | 1.00 | – |
| 143.0–212.0 | 1.25 | (0.92–1.69) | 0.95 | (0.54–1.67) | 1.38 | (0.96–1.98) |
| 212.1–274.0 | 0.96 | (0.69–1.33) | 0.96 | (0.53–1.72) | 0.91 | (0.61–1.36) |
| 274.1–343.1 | 0.83 | (0.60–1.17) | 0.61 | (0.33–1.11) | 0.93 | (0.61–1.41) |
| >343.1 | 0.68 | (0.47–0.97) | 0.45 | (0.24–0.85) | 0.80 | (0.51–1.26) |
| *P*-value for trend | 0.002 | | 0.002 | | 0.09 | |
| Vitamin E (IU day$^{-1}$) | | | | | | |
| ≤5.2 | 1.00 | – | 1.00 | – | 1.00 | – |
| 5.3–6.2 | 0.90 | (0.65–1.24) | 0.86 | (0.47–1.59) | 0.88 | (0.59–1.30) |
| 6.3–7.2 | 0.89 | (0.64–1.26) | 1.00 | (0.53–1.90) | 0.80 | (0.53–1.21) |
| 7.3–8.6 | 0.65 | (0.45–0.95) | 0.68 | (0.35–1.33) | 0.61 | (0.38–0.96) |
| >8.6 | 0.71 | (0.48–1.05) | 0.50 | (0.25–1.02) | 0.85 | (0.53–1.36) |
| *P*-value for trend | 0.04 | | 0.03 | | 0.25 | |

[a]The nutrients in the table are not mutually adjusted; the model includes variables for age (years), birth place (urban vs rural), body mass index (kg m$^{-2}$), parity (parous vs nulliparous), age at first birth (years; among parous women), age at menarche (years), menopausal status (pre- vs postmenopausal), and total energy intake (quintiles). [b]Adjusted for all variables in [a] except for menopausal status. [c]Reference category. [d]Test for trend is from a multivariate model in which nutrient intake is included as an ordinal variable with values 1–5.

reduces arbitrary decisions on the part of the interviewer. Because only 680 complete case–control triplets were available, a matched analysis would unduly restrict the number of available subjects. Furthermore, previous reports found very similar results using conditional and unconditional logistic regression models. Therefore, all subjects were included in the present analysis. In addition, because the comparison of breast cancer cases to either set of controls produced similar results for previously examined exposures, the two control groups were combined to improve the precision of the effect estimates.

A core model was used to control for the potential confounding effects of established demographic and reproductive risk factors for breast cancer. These factors include age (years), place of birth (urban, rural), Quetelet index (kg m$^{-2}$), parity (parous, nulliparous), age at first full-term pregnancy (years; among parous women), age at menarche (years), menopausal status (post-menopausal, premenopausal) and total energy intake (quintiles). Using evenly spaced scores, the trend across the quintile of intake was assessed by a chi-squared test with one degree of freedom. The correlation between pairs of energy-adjusted micronutrients was assessed using Spearman correlation coefficients. In addition, because the effect of some breast cancer risk factors appears to vary by menopausal status, we used the likelihood ratio test to test for interaction between each micronutrient and menopausal status. Women were defined as post-menopausal if they had not had a menstrual period in the previous 12 months, were using hormonal therapy or had had a hysterectomy. Otherwise, women were classified as pre- or perimenopausal.

### RESULTS

The distribution of cases and controls by age, place of birth, body mass index, parity, age at first birth, age at menarche and menopausal status is presented in Table 1. The differences between the case and control distributions are consistent with established breast cancer risk factors, and these variables are included in all subsequent multivariate models.

**Table 4** Correlations between pairs of energy-adjusted micronutrient intakes among the controls

| | Retinol | β-Carotene | Vitamin C | Vitamin E |
|---|---|---|---|---|
| Retinol | | | | |
| β-carotene | 0.003 | | | |
| Vitamin C | –0.08 | 0.49 | | |
| Vitamin E | 0.12 | 0.26 | 0.24 | |

© Cancer Research Campaign 1999 

**Table 5** Multiple logistic regression derived odds ratios (OR) and 95% confidence intervals (CI) for a one-quintile increase in micronutrient intake, without and with mutual adjustment among micronutrients, among all women and stratified by menopausal status

| | All women | | Premenopausal women | | Post-menopausal women | |
|---|---|---|---|---|---|---|
| | OR[a] | 95% CI | OR[b] | 95% CI | OR[b] | 95% CI |
| Without mutual adjustment | | | | | | |
| β-Carotene | 0.90 | (0.84–0.97) | 0.79 | (0.70–0.89) | 0.96 | (0.88–1.05) |
| Vitamin C | 0.88 | (0.81–0.96) | 0.80 | (0.70–0.92) | 0.92 | (0.83–1.01) |
| Vitamin E | 0.91 | (0.83–0.99) | 0.84 | (0.72–0.98) | 0.94 | (0.84–1.05) |
| With mutual adjustment | | | | | | |
| β-Carotene | 0.95 | (0.87–1.03) | 0.84 | (0.73–0.97) | 1.00 | (0.90–1.11) |
| Vitamin C | 0.92 | (0.84–1.01) | 0.90 | (0.77–1.05) | 0.92 | (0.82–1.04) |
| Vitamin E | 0.95 | (0.86–1.04) | 0.93 | (0.79–1.10) | 0.95 | (0.85–1.07) |

[a]The model includes variables for age (years), birth place (urban vs rural), body mass index (kg $m^{-2}$), parity (parous vs nulliparous), age at first birth (years; among parous women), age at menarche (years), menopausal status (pre- vs post-menopausal) and total energy intake (quintile). [b]Adjusted for all variables in [a] except for menopausal status.

The likelihood ratio tests of the interactions between menopausal status and intake of each micronutrient in relation to risk of breast cancer were significant for β-carotene, vitamin C and vitamin E, but not for retinol (data not shown). Therefore, all results are presented separately for pre- and post-menopausal women, as well as for all women combined.

Total energy intake was slightly higher among cases than among controls, but this may reflect over-reporting (which is adjusted for when energy intake is accounted for). Cases consumed an average of 1939 kcal per day, compared with 1905 kcal per day among the controls. When risk of breast cancer by quintile of total energy intake was considered, it was not clear whether log-risk changed in a log-linear manner across the quintiles (data not shown). Therefore, total energy intake was modelled as a categorical, rather than continuous, variable.

Table 2 presents the crude frequency distribution of cases and controls by control-defined quintile of each micronutrient of interest. The multiple logistic regression-derived odds ratios (OR) and 95% confidence intervals (95% CI) by quintile of intake are presented in Table 3. The micronutrients in this table are not mutually adjusted, but are adjusted for total energy intake and the variables in Table 1. Retinol is not associated with risk of breast cancer in either pre- or post-menopausal women. For β-carotene, vitamin C and vitamin E, there are significant inverse trends with increasing intake among premenopausal women only. The strongest inverse association is observed for β-carotene: among premenopausal women, the odds ratio for the highest quintile of β-carotene intake relative to the lowest quintile is 0.36 (95% CI 0.21–0.61), with a *P*-value for the trend across quintiles of 0.0001. Among post-menopausal women, those in the highest quintiles of intake of β-carotene, vitamin C and vitamin E had a non-significant reduction in risk; in no case was the trend across quintiles significant among post-menopausal women. Further adjustment for alcohol intake and use of post-menopausal hormones did not materially alter the results in Table 3, nor did adjustment for total energy intake as a continuous, rather than categorical, variable (data not shown). Differences between the crude associations in Table 2 and the adjusted associations in Table 3 are due to possible confounding of the crude associations, in part by the constellation of risk factors and in part by the higher energy intake among the cases (genuine or, to a certain extent, explained by over-reporting).

Although the results of Table 3 are compatible with a protective role among premenopausal women for three of the four micronutrients evaluated, the correlations among the micronutrients must be considered. Table 4 presents the Spearman correlation coefficients, among controls, for the micronutrients of interest. Beta-carotene and vitamin C are the most highly correlated variables, with $r = 0.49$. Given this degree of correlation, the effect of mutual adjustment among the micronutrients is of interest. Table 5 presents both the non-mutually adjusted and mutually adjusted odds ratios and 95% confidence intervals for a one-quintile increase in intake for β-carotene, vitamin C and vitamin E, among all women and after stratification by menopausal status. Although the effect of each micronutrient is attenuated after mutual adjustment, the effect of β-carotene remains significant among premenopausal women; the odds ratio for a one-quintile increase in intake is 0.84 (95% CI 0.73–0.97). This apparent effect is stronger than those of vitamin C and vitamin E. As before, none of the associations achieved statistical significance among post-menopausal women.

### DISCUSSION

The results of the present analysis suggest an inverse association between breast cancer and β-carotene intake among premenopausal women. Vitamins C and E also appear inversely associated with breast cancer risk among premenopausal women, but the effect of each is substantially attenuated upon adjustment for β-carotene. There was no effect of retinol intake among premenopausal women. Among post-menopausal women, there is some suggestion of an inverse association with each of the micronutrients evaluated, but none of these associations was statistically significant.

Bias and confounding must be considered as possible explanations for the observed results. The documentation in this study of established breast cancer risk factors argues against selection bias. Recall bias is always a concern in case–control studies, but lack of awareness among women in this population of any link between fruit and vegetable intake and risk of breast cancer should minimize this problem. However, the fact that cohort studies generally have observed weaker associations for these nutrients than case–control studies raises the possibility that recall bias may still play a role.

© Cancer Research Campaign 1999

Strengths of the study include the high variability in intake of fruits and vegetables and their component nutrients in the study population, as well as the high absolute intake of these foods. The wide range of intake improves the power to detect effects, whereas the high absolute intake of nutritional factors that may have growth-controlling, rather than initiation-limiting, properties suggests that possible exposure thresholds are likely to be exceeded.

Previous case–control studies generally support an inverse association with carotenoid intake. Of 18 case–control studies, 14 (LaVecchia et al, 1987; Katsouyanni et al, 1988; Rohan et al, 1988; Iscovich et al, 1989; van 't Veer et al, 1990; Graham et al, 1991; Ingram et al, 1991; Lee et al, 1991; Zaridze et al, 1991; London et al, 1992; Levi et al, 1993; Holmberg et al, 1994; Freudenheim et al, 1996; Negri et al, 1996) observed a reduced risk of breast cancer among those in the highest category of carotenoid intake, whereas four (Marubini et al, 1988; Toniolo et al, 1989; Ewertz and Gill, 1990; Richardson et al, 1991) observed no reduction in risk. Of the inverse associations, six (Graham et al, 1991; Lee et al, 1991; Zaridze et al, 1991; Holmberg et al, 1994; Freudenheim et al, 1996; Negri et al, 1996) were statistically significant. A combined analysis of the data from eight case–control studies reported an odds ratio of 0.85 ($P = 0.007$) for the highest versus the lowest quintile of carotenoid intake (Howe et al, 1990). The results from six prospective studies are weaker; four (Paganini-Hill et al, 1987; Graham et al, 1992; Hunter et al, 1993; Rohan et al, 1993) observed inverse but non-significant associations, and two (Kushi et al, 1996; Verhoeven et al, 1997) found no evidence of an inverse association. Although the majority of the case–control and cohort studies did not evaluate specific carotenoids other than β-carotene, one recent case–control study presented results for several carotenoids. Freudenheim et al (1996) report significant inverse associations with β-carotene, α-carotene and lutein–zeaxanthin. The association with lycopene was inverse but not statistically significant, and no association was observed with β-cryptoxanthin.

Although clinical trial data are not yet available regarding the effect of β-carotene supplementation on risk of breast cancer, β-carotene supplementation has been assessed in relation to cancer at other sites. The outcomes of these trials have differed. A trial of β-carotene supplementation in relation to premalignant oral lesions suggested that β-carotene may promote regression of the lesions (Sankaranarayanan et al, 1997). Two other trials, primarily among smokers, reported an increased risk of lung cancer among those receiving β-carotene (The Alpha-Tocopherol, Beta-Carotene Cancer Prevention Study Group, 1994) or β-carotene in combination with retinyl palmitate (Omenn et al, 1996). A third trial found no association between β-carotene supplementation and risk of lung cancer or other malignant neoplasms (Hennekens et al, 1996). Two trials of β-carotene supplementation in relation to recurrent skin cancer (Greenberg et al, 1990) and colon polyps (Greenberg et al, 1994) also reported no association.

Of 13 case–control studies to evaluate vitamin C intake, eight (Iscovich et al, 1989; Graham et al, 1991; Zaridze et al, 1991; Levi et al, 1993; Landa et al, 1994; Yuan et al, 1995; Freudenheim et al, 1996; Negri et al, 1996) report odds ratios of less than 0.8, and five (Graham et al, 1982; Katsouyanni et al, 1988; Toniolo et al, 1989; Ingram et al, 1991; Holmberg et al, 1994) report odds ratios greater than or equal to 1.0. Howe et al (1990), in a combined analysis of the data from nine case–control studies, reported an odds ratio of 0.69 ($P<0.0001$) for the highest quintile of vitamin C intake. Three prospective studies (Graham et al, 1992; Rohan et al, 1993; Verhoeven et al, 1997) observed relative risks of less than 0.90, although another two (Hunter et al, 1993; Kushi et al, 1996) did not. None of the results from prospective studies were statistically significant.

Fewer studies have evaluated vitamin E intake. Of ten case–control studies, seven (Graham et al, 1991; Lee et al, 1991; London et al, 1992; Holmberg et al, 1994; Yuan et al, 1995; Freudenheim et al, 1996; Negri et al, 1996) suggest an inverse association with vitamin E intake and three (Toniolo et al, 1989; Gerber et al, 1990; Richardson et al, 1991) report null or positive associations; the results are statistically significant in four (Graham et al, 1991; London et al, 1992; Freudenheim et al, 1996; Negri et al, 1996) of the studies reporting inverse associations. The results from cohort studies are decidedly weaker: of five studies (Graham et al, 1992; Hunter et al, 1993; Rohan et al, 1993; Kushi et al, 1996; Verhoeven et al, 1997), only one (Graham et al, 1992) reports a relative risk of less than 0.90, and this was not statistically significant.

A handful of studies report a protective association with retinol, but the overall picture is one of a null or even positive association with breast cancer. Of 13 case–control studies, six (La Vecchia et al, 1987; Katsouyanni et al, 1988; Marubini et al, 1988; Zaridze et al, 1991; London et al, 1992; Negri et al, 1996) report inverse associations, and seven (Rohan et al, 1988; Toniolo et al, 1989; Graham et al, 1991; Ingram et al, 1991; Richardson et al, 1991; Levi et al, 1993; Yuan et al, 1995) report null or positive findings. Of the case–control studies reporting inverse associations, only one (Negri et al, 1996) approaches statistical significance at the highest category of intake. Of the case–control studies reporting null or positive associations, Richardson et al (1991) report a statistically significant increased risk at the highest category of intake among post-menopausal women (OR = 2.8, 95% CI 1.2–2.8). In a combined analysis of seven case–control studies, Howe et al (1990) report an odds ratio of 1.04 for the highest quintile of retinol intake. Of five prospective studies, two (Hunter et al, 1993; Rohan et al, 1993) report inverse associations with retinol intake, and three (Graham et al, 1992; Kushi et al, 1996; Verhoeven et al, 1997) report null or positive associations. The only statistically significant finding from a prospective study is reported by Hunter et al (1993), in which the relative risk for the highest quintile of intake is 0.80 (95% CI 0.68–0.95).

Plausible biological mechanisms by which each of these micronutrients may affect breast cancer risk do exist, although none of these mechanisms has been shown to be of importance in the development of breast cancer in humans. β-carotene and other carotenoids are potent antioxidants (Peto et al, 1981; Ames, 1983) and may protect against free radical-induced DNA damage. Vitamin E is a lipid-soluble antioxidant that protects against lipid peroxidation of cell membranes (Ames, 1983). Vitamin C is a water-soluble antioxidant that may also play a role in immune function and in the sparing of other antioxidants, including vitamin E (Block, 1991). With the exception of retinol, results for each of the micronutrients assessed were more strongly inversely associated with breast cancer risk among premenopausal than among post-menopausal women. The reasons for this are not clear, but the proximity of younger women to the aetiologically relevant time period is a possible explanation. Several previous studies have considered differences by menopausal status in the effect of micronutrients (La Vecchia et al, 1987; Katsouyanni et al, 1988; Marubini et al, 1988; Rohan et al, 1988; Howe et al, 1990; Richardson et al, 1991; Zaridze et al, 1991; Lee et al, 1992; Hunter

© Cancer Research Campaign 1999

et al, 1993; Rohan et al, 1993; Yuan et al, 1995), but with inconsistent results. Approximately half of all studies reporting results by menopausal status indicate no differences in pre- and post-menopausal women (La Vecchia et al, 1987; Katsouyanni et al, 1988; Marubini et al, 1988; Rohan et al, 1993; Yuan et al, 1995). Of studies that found even a modest difference by menopausal status, one (Zaridze et al, 1991) reported stronger protective effects among post-menopausal women, and three (Rohan et al, 1988; Lee et al, 1992; Hunter et al, 1993) reported stronger protective effects among premenopausal women for at least one nutrient. Howe et al (1990), in a combined analysis of the data from 12 case–control studies, reported that the protective effects of β-carotene and vitamin C were stronger among post-menopausal women than among premenopausal women, although the tests for heterogeneity were not significant. In light of these inconsistencies, and without a strong basis for the apparent effect modification, the differences by menopausal status observed in the present study should be interpreted cautiously.

Although the current and several previous studies have found an inverse association between β-carotene and breast cancer, one must consider the extent to which the observed effect is because of the nutrient per se, and the extent to which it can be explained by the correlation of the nutrient with other unmeasured components of fruits and vegetables. Several previous studies, reviewed by Potter and Steinmetz (1996), have indicated that high intake of fruits and vegetables may be associated with a reduced risk of breast cancer. If the protection conferred by fruits and vegetables is attributable to one of the micronutrients evaluated, the absolute value of the effect estimate for the micronutrient (per quintile or other centile) should be larger than the corresponding estimate for fruits and vegetables. In a previously published report from this study, the logistic regression parameter estimate for a one-quintile increase in vegetable intake was –0.1374 (Trichopoulou et al, 1995), whereas in the current analysis the estimate for a one-quintile increase in β-carotene intake is a weaker –0.1014. Definitive conclusions, however, cannot be drawn from these findings. First, β-carotene intake is measured with greater error than fruit and vegetable intake, and the resulting attenuation of the β-carotene effect may mask a stronger effect. It, therefore, may not be possible to rule out β-carotene as a principal protective component of fruits and vegetables. Second, the weaker effect of β-carotene may indicate that β-carotene is only one of many micronutrients that contribute to the protection conferred by vegetable intake. Third and final, β-carotene may have no effect on breast cancer risk; the observed protective effect may be explained entirely by another component of fruits and vegetables that is correlated with β-carotene intake. A variety of such substances are discussed in a review by Steinmetz and Potter (1991). Given these various possibilities, it would be premature to consider β-carotene as playing a major role in reducing breast cancer risk.

Although the effect of vitamin E was not statistically significant, it was clearly inverse. In this population of Greek women, olive oil is an important source of vitamin E. A previously published report from this study demonstrated a statistically significant inverse association between olive oil intake and risk of breast cancer (Trichopoulou et al, 1995). This association is stronger than that of vitamin E, suggesting that vitamin E itself may not be the sole explanation for the apparent protection conferred by olive oil.

In conclusion, the present analysis did not demonstrate a strong independent effect on breast cancer risk of any of the micronutrients evaluated. A protective effect of β-carotene intake was observed among premenopausal women, but it is not possible to rule out the possibility that this apparent effect is due, at least in part, to other components of fruits and vegetables. In light of these data, the most prudent public health message would be to increase intake of fruits and vegetables. Supplementation with β-carotene, although probably not harmful, has no clear indication in the context of breast cancer risk.

### REFERENCES

Albanes D (1987) Total calories, body weight, and tumor incidence in mice. *Cancer Res* **47**: 1987–1992

Ames BN (1983) Dietary carcinogens and anticarcinogens. Oxygen radicals and degenerative diseases. *Science* **221**: 1256–1264

Block G (1991) Vitamin C and cancer prevention: the epidemiologic evidence. *Am J Clin Nutr* **53**: 270S–282S

Breslow NE and Day NE (1980) The analysis of case–control studies. In *Statistical Methods in Cancer Research*. Vol. 1. IARC scientific publication no. 32. International Agency for Research on Cancer: Lyon

Buell P (1973) Changing incidence of breast cancer in Japanese–American women. *J Natl Cancer Inst* **51**: 1479–1483

Ewertz M and Gill C (1990) Dietary factors and breast cancer risk in Denmark. *Int J Cancer* **46**: 779–784

Freudenheim JL, Marshall JR, Vena JE, Laughlin R, Brasure JR, Swanson MK, Nemoto T and Graham S (1996) Premenopausal breast cancer risk and intake of vegetables, fruits, and related nutrients. *J Natl Cancer Inst* **88**: 340–348

Gerber M, Richardson S, Cavallo F, Marubini E, Crastes de Paulet P, Crastes de Paulet A and Pujol H (1990) The role of diet history and biologic assays in the study of ‘diet and breast cancer’. *Tumori* **76**: 321–330

Gnardellis C, Trichopoulou A, Katsouyanni K, Polychronopoulos E, Rimm EB and Trichopoulos D (1995) Reproducibility and validity of an extensive semiquantitative food frequency questionnaire among Greek school teachers. *Epidemiology* **6**: 74–77

Graham S, Dayal H, Swanson M, Mittelman A and Wilkinson G (1978) Diet in the epidemiology of cancer of the colon and rectum. *J Natl Cancer Inst* **61**: 709–714

Graham S, Marshall J, Mettlin C, Rzepka T, Nemoto T and Byers T (1982) Diet in the epidemiology of breast cancer. *Am J Epidemiol* **116**: 68–75

Graham S, Hellmann R, Marshall J, Freudenheim J, Vena J, Swanson M, Zielezny M, Nemoto T, Stubbe N and Raimondo T (1991) Nutritional epidemiology of postmenopausal breast cancer in western New York. *Am J Epidemiol* **134**: 552–566

Graham S, Zielezny M, Marshall J, Priore R, Freudenheim J, Brasure J, Haughey B, Nasca P and Zdeb M (1992) Diet in the epidemiology of postmenopausal breast cancer in the New York State Cohort. *Am J Epidemiol* **136**: 1327–1337

Greenberg ER, Baron JA, Stukel TA, Stevens MM, Mandel JS, Spencer SK, Elias PM, Lowe N, Nierenberg DW, Bayrd G, Vance JC, Freeman DH, Clendenning WE, Kwan T and the Skin Cancer Prevention Study Group (1990) A clinical trial of beta carotene to prevent basal-cell and squamous-cell cancers of the skin. *N Engl J Med* **323**: 789–795. [Erratum, *N Engl J Med* (1991) **325**: 1324]

Greenberg ER, Baron JA, Tosteson TD, Freeman DH, Beck GJ, Bond JH, Colacchio TA, Coller JA, Frankl HD, Haile RW, Mandel JS, Nierenberg DW, Rothstein R, Snover DC, Stevens MM, Summers RW and van Stolk RU for the Polyp Prevention Study Group (1994) A clinical trial of antioxidant vitamins to prevent colorectal adenoma. *N Engl J Med* **331**: 141–147

Hennekens CH, Buring JE, Manson JE, Stampfer M, Rosner B, Cook NR, Belanger C, LaMotte F, Gaziano JM, Ridker PM, Willett W and Peto R (1996) Lack of effect of long-term supplementation with beta carotene on the incidence of malignant neoplasms and cardiovascular disease. *N Engl J Med* **334**: 1145–1149

Holmberg L, Ohlander EM, Byers T, Zack M, Wolk A, Bergstrom R, Bergkvist L, Thurfjell E, Bruce A and Adami H-O (1994) Diet and breast cancer risk: results from a population-based case–control study in Sweden. *Arch Intern Med* **154**: 1805–1811

Howe GR, Hirohata T, Hislop TG, Iscovich JM, Yuan J-M, Katsouyanni K, Lubin F, Marubini E, Modan B, Rohan T, Toniolo P and Shunzhang Y (1990) Dietary factors and risk of breast cancer: combined analysis of 12 case–control studies. *J Natl Cancer Inst* **82**: 561–569

Hunter DJ and Willett WC (1996) Nutrition and breast cancer. *Cancer Causes Control* **7**: 56–68

Hunter DJ, Manson JE, Colditz GA, Stampfer MJ, Rosner B, Hennekens CH, Speizer FE and Willett WC (1993) A prospective study of the intake of vitamins C, E, and A and the risk of breast cancer. *N Engl J Med* **329**: 234–240

© Cancer Research Campaign 1999

Ingram DM, Nottage E and Roberts T (1991) The role of diet in the development of breast cancer: a case–control study of patients with breast cancer, benign epithelial hyperplasia and fibrocystic disease of the breast. *Br J Cancer* **64**: 187–191

Iscovich JM, Iscovich RB, Howe G, Shiboski S and Kaldor JM (1989) A case–control study of diet and breast cancer in Argentina. *Int J Cancer* **44**: 770–776

Katsouyanni K, Willett W, Trichopoulos D, Boyle P, Trichopoulou A, Vasilaros S, Papadiamantis J and MacMahon B (1988) Risk of breast cancer among Greek women in relation to nutrient intake. *Cancer* **61**: 181–185

Katsouyanni K, Skalkidis Y, Petridou E, Polychronopoulou-Trichopoulou A, Willett W and Trichopoulos D (1991*a*) Diet and peripheral arterial occlusive disease: the role of poly-, mono-, and saturated fatty acids. *Am J Epidemiol* **133**: 24–31

Katsouyanni K, Trichopoulou A, Trichopoulos D and Willett W (1991*b*) *Dietary Variability in Greece. A Report to the Secretaria of Research and Technology*, pp. 1–64. Ministery of Industry, Research and Technology: Athens

Katsouyanni K, Trichopoulou A, Stuver S, Garas Y, Kritselis A, Kyriakou G, Stoikidou M, Boyle P and Trichopoulos D (1994*a*) The association of fat and other macronutrients with breast cancer: a case–control study from Greece. *Br J Cancer* **70**: 537–541

Katsouyanni K, Trichopoulou A, Stuver S, Vassilaros S, Papadiamantis Y, Bournas N, Skarpou N, Mueller N and Trichopoulos D (1994*b*) Ethanol and breast cancer: an association that may be both confounded and causal. *Int J Cancer* **58**: 356–361

Katsouyanni K, Rimm EB, Gnardellis C, Trichopoulos D, Polychronopoulos E and Trichopoulou A (1997) Reproducibility and relative validity of an extensive semi-quantitative food frequency questionnaire using dietary records and biochemical markers among Greek schoolteachers. *Int J Epidemiol* **26** (suppl. 1): S118–S127

Kushi LH, Fee RM, Sellers TA, Zheng W and Folsom AR (1996) Intake of vitamins A, C, and E and postmenopausal breast cancer: the Iowa Women's Health Study. *Am J Epidemiol* **144**: 165–174

Landa M-C, Frago N and Tres A (1994) Diet and the risk of breast cancer in Spain. *Eur J Cancer Prev* **3**: 313–320

La Vecchia C, Decarli A, Franceschi S, Gentile A, Negri E and Parazzini F (1987) Dietary factors and the risk of breast cancer. *Nutr Cancer* **10**: 205–214

Lee HP, Gourley L, Duffy SW, Esteve J, Lee J and Day NE (1991) Dietary effects on breast-cancer risk in Singapore. *Lancet* **337**: 1197–1200

Lee HP, Gourley L, Duffy SW, Esteve J, Lee J and Day NE (1992) Risk factors for breast cancer by age and menopausal status: a case–control study in Singapore. *Cancer Causes Control* **3**: 313–322

Levi F, La Vecchia C, Gulie C and Negri E (1993) Dietary factors and breast cancer risk in Vaud, Switzerland. *Nutr Cancer* **19**: 327–335

London SJ, Stein EA, Henderson IC, Stampfer MJ, Wood WC, Remine S, Dmochowski JR, Robert NJ and Willett WC (1992) Carotenoids, retinol, and vitamin E and risk of proliferative benign breast disease and breast cancer. *Cancer Causes Control* **3**: 503–512

Marubini E, Decarli A, Costa A, Mazzoleni C, Andreoli C, Barbieri A, Capitelli E, Carlucci M, Cavallo F, Monferroni N, Pastorino U and Salvini S (1988) The relationship of dietary intake and serum levels of retinol and beta-carotene with breast cancer: results of a case–control study. *Cancer* **61**: 173–180

Negri E, La Vecchia C, Franceschi S, D'Avanzo B, Talamini R, Parpinel M, Ferraroni M, Filiberti R, Montella M, Falcini F, Conti E and Decarli A (1996) Intake of selected micronutrients and the risk of breast cancer. *Int J Cancer* **65**: 140–144

Omenn GS, Goodman GE, Thornquist MD, Balmes J, Cullen MR, Glass A, Keogh JP, Meyskens FL, Valanis B, Williams JH, Barnhart S and Hammar S (1996) Effects of a combination of beta carotene and vitamin A on lung cancer and cardiovascular disease. *N Engl J Med* **334**: 1150–1155

Paganini-Hill A, Chao A, Ross RK and Henderson BE (1987) Vitamin A, β-carotene, and the risk of cancer: a prospective study. *J Natl Cancer Inst* **79**: 443–448

Peto R, Doll R, Buckley JD and Sporn MB (1981) Can dietary beta-carotene materially reduce human cancer rates? *Nature* **290**: 201–208

Potter JD and Steinmetz K (1996) Vegetables, fruit and phytoestrogens as preventive agents. In *Principles of Chemoprevention*. Stewart BW, McGregor D and Kleihues P (eds), pp. 61–90. IARC Scientific Publications no. 139. International Agency for Research on Cancer: Lyon

Richardson S, Gerber M and Cenee S (1991) The role of fat, animal protein and some vitamin consumption in breast cancer: a case–control study in southern France. *Int J Cancer* **48**: 1–9

Rohan TE, McMichael AJ and Baghurst PA (1988) A population-based case–control study of diet and breast cancer in Australia. *Am J Epidemiol* **128**: 478–489

Rohan TE, Howe GR, Friedenreich CM, Jain M and Miller AB (1993) Dietary fiber, vitamins A, C, and E, and risk of breast cancer: a cohort study. *Cancer Causes Control* **4**: 29–37

Sankaranarayanan R, Mathew B, Varghese C, Sudhakaran PR, Menon V, Jayadeep A, Nair MK, Mathews C, Mahalingam TR, Balaram P and Nair PP (1997) Chemoprevention of oral leukoplakia with vitamin A and beta carotene: an assessment. *Oral Oncol* **33**: 231–236

Shimizu H, Ross RK, Bernstein L, Yatani R, Henderson BE and Mack TM (1991) Cancers of the prostate and breast among Japanese and white immigrants in Los Angeles County. *Br J Cancer* **63**: 963–966

Sporn MB and Roberts AB (1983) Role of retinoids in differentiation and carcinogenesis. *Cancer Res* **43**: 3034–3040

Steinmetz KA and Potter JD (1991) Vegetables, fruit, and cancer. II. Mechanisms. *Cancer Causes Control* **2**: 427–442

Tannenbaum A (1942) The genesis and growth of tumors. III. Effects of a high-fat diet. *Cancer Res* **2**: 468–475

The Alpha-Tocopherol, Beta-Carotene Cancer Prevention Study Group (1994) The effect of vitamin E and beta carotene on the incidence of lung cancer and other cancers in male smokers. *N Engl J Med* **330**: 1029–1035

Thomas DB and Karagas MR (1987) Cancer in first and second generation Americans. *Cancer Res* **47**: 5771–5776

Toniolo P, Riboli E, Protta F, Charrel M and Cappa APM (1989) Calorie-providing nutrients and risk of breast cancer. *J Natl Cancer Inst* **81**: 278–286

Trichopoulou A (1992) *Composition of Greek Foods and Dishes* (in Greek and English). Athens School of Public Health: Athens

Trichopoulou A, Katsouyanni K, Stuver S, Tzala L, Gnardellis C, Rimm E and Trichopoulos D (1995) Consumption of olive oil and specific food groups in relation to breast cancer risk in Greece. *J Natl Cancer Inst* **87**: 110–116

van 't Veer P, Kolb CM, Verhoef P, Kok FJ, Schouten EG, Hermus RJJ and Sturmans F (1990) Dietary fiber, beta-carotene and breast cancer: results from a case–control study. *Int J Cancer* **45**: 825–828

Verhoeven DTH, Assen N, Goldbohm RA, Dorant E, van 't Veer P, Sturmans F, Hermus RJJ and van den Brandt PA (1997) Vitamins C and E, retinol, beta-carotene and dietary fibre in relation to breast cancer risk: a prospective cohort study. *Br J Cancer* **75**: 149–155

Yuan J-M, Wang Q-S, Ross RK, Henderson BE and Yu MC (1995) Diet and breast cancer in Shanghai and Tianjin, China. *Br J Cancer* **71**: 1353–1358

Zaridze D, Lifanova Y, Maximovitch D, Day NE and Duffy SW (1991) Diet, alcohol consumption and reproductive factors in a case–control study of breast cancer in Moscow. *Int J Cancer* **48**: 493–501

Ziegler RG, Hoover RN, Pike MC, Hildesheim A, Nomura AMY, West DW, Wu-Williams AH, Kolonel LN, Horn-Ross PL, Rosenthal JF and Hyer MB (1993) Migration patterns and breast cancer risk in Asian–American women. *J Natl Cancer Inst* **85**: 1819–1827

*© Cancer Research Campaign 1999*

## References

[PDF_00537] Albanes D. (1987). Total calories, body weight, and tumor incidence in mice.. Cancer Res.

[PDF_00539] Ames B. N. (1983). Dietary carcinogens and anticarcinogens. Oxygen radicals and degenerative diseases.. Science.

[PDF_00541] Block G. (1991). Vitamin C and cancer prevention: the epidemiologic evidence.. Am J Clin Nutr.

[PDF_00546] Buell P. (1973). Changing incidence of breast cancer in Japanese-American women.. J Natl Cancer Inst.

[PDF_00548] Ewertz M., Gill C. (1990). Dietary factors and breast-cancer risk in Denmark.. Int J Cancer.

[PDF_00550] Freudenheim J. L., Marshall J. R., Vena J. E., Laughlin R., Brasure J. R., Swanson M. K., Nemoto T., Graham S. (1996). Premenopausal breast cancer risk and intake of vegetables, fruits, and related nutrients.. J Natl Cancer Inst.

[PDF_00553] Gerber M., Richardson S., Cavallo F., Marubini E., Crastes de Paulet P., Crastes de Paulet A., Pujol H. (1990). The role of diet history and biologic assays in the study of "diet and breast cancer".. Tumori.

[PDF_00556] Gnardellis C., Trichopoulou A., Katsouyanni K., Polychronopoulos E., Rimm E. B., Trichopoulos D. (1995). Reproducibility and validity of an extensive semiquantitative food frequency questionnaire among Greek school teachers.. Epidemiology.

[PDF_00560] Graham S., Dayal H., Swanson M., Mittelman A., Wilkinson G. (1978). Diet in the epidemiology of cancer of the colon and rectum.. J Natl Cancer Inst.

[PDF_00565] Graham S., Hellmann R., Marshall J., Freudenheim J., Vena J., Swanson M., Zielezny M., Nemoto T., Stubbe N., Raimondo T. (1991). Nutritional epidemiology of postmenopausal breast cancer in western New York.. Am J Epidemiol.

[PDF_00563] Graham S., Marshall J., Mettlin C., Rzepka T., Nemoto T., Byers T. (1982). Diet in the epidemiology of breast cancer.. Am J Epidemiol.

[PDF_00569] Graham S., Zielezny M., Marshall J., Priore R., Freudenheim J., Brasure J., Haughey B., Nasca P., Zdeb M. (1992). Diet in the epidemiology of postmenopausal breast cancer in the New York State Cohort.. Am J Epidemiol.

[PDF_00572] Greenberg E. R., Baron J. A., Stukel T. A., Stevens M. M., Mandel J. S., Spencer S. K., Elias P. M., Lowe N., Nierenberg D. W., Bayrd G. (1990). A clinical trial of beta carotene to prevent basal-cell and squamous-cell cancers of the skin. The Skin Cancer Prevention Study Group.. N Engl J Med.

[PDF_00577] Greenberg E. R., Baron J. A., Tosteson T. D., Freeman D. H., Beck G. J., Bond J. H., Colacchio T. A., Coller J. A., Frankl H. D., Haile R. W. (1994). A clinical trial of antioxidant vitamins to prevent colorectal adenoma. Polyp Prevention Study Group.. N Engl J Med.

[PDF_00582] Hennekens C. H., Buring J. E., Manson J. E., Stampfer M., Rosner B., Cook N. R., Belanger C., LaMotte F., Gaziano J. M., Ridker P. M. (1996). Lack of effect of long-term supplementation with beta carotene on the incidence of malignant neoplasms and cardiovascular disease.. N Engl J Med.

[PDF_00587] Holmberg L., Ohlander E. M., Byers T., Zack M., Wolk A., Bergström R., Bergkvist L., Thurfjell E., Bruce A., Adami H. O. (1994). Diet and breast cancer risk. Results from a population-based, case-control study in Sweden.. Arch Intern Med.

[PDF_00591] Howe G. R., Hirohata T., Hislop T. G., Iscovich J. M., Yuan J. M., Katsouyanni K., Lubin F., Marubini E., Modan B., Rohan T. (1990). Dietary factors and risk of breast cancer: combined analysis of 12 case-control studies.. J Natl Cancer Inst.

[PDF_00597] Hunter D. J., Manson J. E., Colditz G. A., Stampfer M. J., Rosner B., Hennekens C. H., Speizer F. E., Willett W. C. (1993). A prospective study of the intake of vitamins C, E, and A and the risk of breast cancer.. N Engl J Med.

[PDF_00595] Hunter D. J., Willett W. C. (1996). Nutrition and breast cancer.. Cancer Causes Control.

[PDF_00602] Ingram D. M., Nottage E., Roberts T. (1991). The role of diet in the development of breast cancer: a case-control study of patients with breast cancer, benign epithelial hyperplasia and fibrocystic disease of the breast.. Br J Cancer.

[PDF_00606] Iscovich J. M., Iscovich R. B., Howe G., Shiboski S., Kaldor J. M. (1989). A case-control study of diet and breast cancer in Argentina.. Int J Cancer.

[PDF_00626] Katsouyanni K., Rimm E. B., Gnardellis C., Trichopoulos D., Polychronopoulos E., Trichopoulou A. (1997). Reproducibility and relative validity of an extensive semi-quantitative food frequency questionnaire using dietary records and biochemical markers among Greek schoolteachers.. Int J Epidemiol.

[PDF_00612] Katsouyanni K., Skalkidis Y., Petridou E., Polychronopoulou-Trichopoulou A., Willett W., Trichopoulos D. (1991). Diet and peripheral arterial occlusive disease: the role of poly-, mono-, and saturated fatty acids.. Am J Epidemiol.

[PDF_00618] Katsouyanni K., Trichopoulou A., Stuver S., Garas Y., Kritselis A., Kyriakou G., Stoïkidou M., Boyle P., Trichopoulos D. (1994). The association of fat and other macronutrients with breast cancer: a case-control study from Greece.. Br J Cancer.

[PDF_00622] Katsouyanni K., Trichopoulou A., Stuver S., Vassilaros S., Papadiamantis Y., Bournas N., Skarpou N., Mueller N., Trichopoulos D. (1994). Ethanol and breast cancer: an association that may be both confounded and causal.. Int J Cancer.

[PDF_00609] Katsouyanni K., Willett W., Trichopoulos D., Boyle P., Trichopoulou A., Vasilaros S., Papadiamantis J., MacMahon B. (1988). Risk of breast cancer among Greek women in relation to nutrient intake.. Cancer.

[PDF_00631] Kushi L. H., Fee R. M., Sellers T. A., Zheng W., Folsom A. R. (1996). Intake of vitamins A, C, and E and postmenopausal breast cancer. The Iowa Women's Health Study.. Am J Epidemiol.

[PDF_00636] La Vecchia C., Decarli A., Franceschi S., Gentile A., Negri E., Parazzini F. (1987). Dietary factors and the risk of breast cancer.. Nutr Cancer.

[PDF_00634] Landa M. C., Frago N., Tres A. (1994). Diet and the risk of breast cancer in Spain.. Eur J Cancer Prev.

[PDF_00640] Lee H. P., Gourley L., Duffy S. W., Estève J., Lee J., Day N. E. (1992). Risk factors for breast cancer by age and menopausal status: a case-control study in Singapore.. Cancer Causes Control.

[PDF_00638] Lee H. P., Gourley L., Duffy S. W., Estéve J., Lee J., Day N. E. (1991). Dietary effects on breast-cancer risk in Singapore.. Lancet.

[PDF_00643] Levi F., La Vecchia C., Gulie C., Negri E. (1993). Dietary factors and breast cancer risk in Vaud, Switzerland.. Nutr Cancer.

[PDF_00645] London S. J., Stein E. A., Henderson I. C., Stampfer M. J., Wood W. C., Remine S., Dmochowski J. R., Robert N. J., Willett W. C. (1992). Carotenoids, retinol, and vitamin E and risk of proliferative benign breast disease and breast cancer.. Cancer Causes Control.

[PDF_00649] Marubini E., Decarli A., Costa A., Mazzoleni C., Andreoli C., Barbieri A., Capitelli E., Carlucci M., Cavallo F., Monferroni N. (1988). The relationship of dietary intake and serum levels of retinol and beta-carotene with breast cancer. Results of a case-control study.. Cancer.

[PDF_00653] Negri E., La Vecchia C., Franceschi S., D'Avanzo B., Talamini R., Parpinel M., Ferraroni M., Filiberti R., Montella M., Falcini F. (1996). Intake of selected micronutrients and the risk of breast cancer.. Int J Cancer.

[PDF_00657] Omenn G. S., Goodman G. E., Thornquist M. D., Balmes J., Cullen M. R., Glass A., Keogh J. P., Meyskens F. L., Valanis B., Williams J. H. (1996). Effects of a combination of beta carotene and vitamin A on lung cancer and cardiovascular disease.. N Engl J Med.

[PDF_00661] Paganini-Hill A., Chao A., Ross R. K., Henderson B. E. (1987). Vitamin A, beta-carotene, and the risk of cancer: a prospective study.. J Natl Cancer Inst.

[PDF_00664] Peto R., Doll R., Buckley J. D., Sporn M. B. (1981). Can dietary beta-carotene materially reduce human cancer rates?. Nature.

[PDF_00666] Potter J. D., Steinmetz K. (1996). Vegetables, fruit and phytoestrogens as preventive agents.. IARC Sci Publ.

[PDF_00670] Richardson S., Gerber M., Cenée S. (1991). The role of fat, animal protein and some vitamin consumption in breast cancer: a case control study in southern France.. Int J Cancer.

[PDF_00675] Rohan T. E., Howe G. R., Friedenreich C. M., Jain M., Miller A. B. (1993). Dietary fiber, vitamins A, C, and E, and risk of breast cancer: a cohort study.. Cancer Causes Control.

[PDF_00673] Rohan T. E., McMichael A. J., Baghurst P. A. (1988). A population-based case-control study of diet and breast cancer in Australia.. Am J Epidemiol.

[PDF_00678] Sankaranarayanan R., Mathew B., Varghese C., Sudhakaran P. R., Menon V., Jayadeep A., Nair M. K., Mathews C., Mahalingam T. R., Balaram P. (1997). Chemoprevention of oral leukoplakia with vitamin A and beta carotene: an assessment.. Oral Oncol.

[PDF_00682] Shimizu H., Ross R. K., Bernstein L., Yatani R., Henderson B. E., Mack T. M. (1991). Cancers of the prostate and breast among Japanese and white immigrants in Los Angeles County.. Br J Cancer.

[PDF_00685] Sporn M. B., Roberts A. B. (1983). Role of retinoids in differentiation and carcinogenesis.. Cancer Res.

[PDF_00687] Steinmetz K. A., Potter J. D. (1991). Vegetables, fruit, and cancer. II. Mechanisms.. Cancer Causes Control.

[PDF_00694] Thomas D. B., Karagas M. R. (1987). Cancer in first and second generation Americans.. Cancer Res.

[PDF_00696] Toniolo P., Riboli E., Protta F., Charrel M., Cappa A. P. (1989). Calorie-providing nutrients and risk of breast cancer.. J Natl Cancer Inst.

[PDF_00698] Trichopoulou A., Katsouyanni K., Stuver S., Tzala L., Gnardellis C., Rimm E., Trichopoulos D. (1995). Consumption of olive oil and specific food groups in relation to breast cancer risk in Greece.. J Natl Cancer Inst.

[PDF_00703] Van 't Veer P., Kolb C. M., Verhoef P., Kok F. J., Schouten E. G., Hermus R. J., Sturmans F. (1990). Dietary fiber, beta-carotene and breast cancer: results from a case-control study.. Int J Cancer.

[PDF_00707] Verhoeven D. T., Assen N., Goldbohm R. A., Dorant E., van 't Veer P., Sturmans F., Hermus R. J., van den Brandt P. A. (1997). Vitamins C and E, retinol, beta-carotene and dietary fibre in relation to breast cancer risk: a prospective cohort study.. Br J Cancer.

[PDF_00710] Yuan J. M., Wang Q. S., Ross R. K., Henderson B. E., Yu M. C. (1995). Diet and breast cancer in Shanghai and Tianjin, China.. Br J Cancer.

[PDF_00712] Zaridze D., Lifanova Y., Maximovitch D., Day N. E., Duffy S. W. (1991). Diet, alcohol consumption and reproductive factors in a case-control study of breast cancer in Moscow.. Int J Cancer.

[PDF_00716] Ziegler R. G., Hoover R. N., Pike M. C., Hildesheim A., Nomura A. M., West D. W., Wu-Williams A. H., Kolonel L. N., Horn-Ross P. L., Rosenthal J. F. (1993). Migration patterns and breast cancer risk in Asian-American women.. J Natl Cancer Inst.

